# Death of M. Ribes

**Published:** 1845-06

**Authors:** 


### OBITUARY.

*M. Ribes.*—M. Ribes was one of the most talented and one of the most celebrated of the French military surgeons of the revolution, and of the empire. Born in 1766, at Bagnères, he first studied at Toulouse under Alexis Larrey, and then at Paris under Desault, Pinel, Sabatier, &c. He was the friend and fellow-student of Bichat, Richerand, Bretonneau, and of many other illustrious men of the present century. In 1792 he adopted the career of military surgery, and, on the recommendation of Sabatier, obtained an appointment at the Hôtel des Invalides. The following year he was drafted into the army. From this epoch, until the definitive return of the Bourbons, he was continually in the field, and assisted at nearly all the campaigns of that eventful period. During his lengthened military career he was present at twenty drawn battles, at seventeen smaller encounters, and at three sieges. He also took an active part in two disastrous epidemics of typhus, which raged, the one in Spain, and the other in Saxony; and he did not even escape participating in the fatal campaign of Russia in 1813. In the course of these numerous campaigns, the dangers which he encountered, the narrow escapes which he experienced, would alone fill a volume. Thus we may mention, that he was standing by the side of General Dugommier, of Marshal Duroc, and of General Kirschner, when, at different periods, they were killed, the first by a shell, the two latter by cannon-balls.

After the peace, Ribes returned to Paris, and devoted his leisure to the scientific study of his profession. It was not, however, till much later, when he had matured his extensive experience by reflection and study, that he submitted to the world those labors which gained him, in the world of letters, the reputation of an eminent scientific surgeon, as well as that which he already possesses, of an able practitioner. His principal work is one in three volumes, which he dedicated to Bichat, entitled "Memoirs and Observations of Anatomy, Physiology, and Surgery." He has also written, in the medical journals of the day, a great number of papers on various subjects.

Ribes was an extremely modest, retiring man, and lived away from the world, in the retirement of private life. At the latter part of his career he was placed at the head of the medical staff of the Invalides, the magnificent military hospital where he had held his first appointment more than half a century before. Here he found himself, to his great delight, amongst his old companions in arms. This appointment was, however, withdrawn from him a short time ago. The blow was a severe one; indeed, he never recovered it. His health gradually declined, and on the 21st of last February, he died of a chronic inflammatory affection of the lungs. He was, at his death, in his eightieth year. Although so advanced in life, until within a short period of his death, he enjoyed all the physical and intellectual activity of a green old age, not a very common occurrence with members of our profession.—*Ibid.*

